# Colon Capsule Endoscopy as a Promising Diagnostic Tool in Colorectal Cancer: A Systematic Review and Network Meta-Analysis

**DOI:** 10.3390/diagnostics15172157

**Published:** 2025-08-26

**Authors:** Emma Altobelli, Paolo Matteo Angeletti, Paolo Angelo Varesini, Zuleyka Bianchi, Francesco Masedu

**Affiliations:** 1 Section of Epidemiology and Public Health Unit, Department of Life, Health and Environmental Sciences, University of L’Aquila, Piazzale Salvatore Tommasi 1, Coppito, 67100 L’Aquila, Italy; 2 Cardiovascular Department, UO of Cardiac Anesthesia of the IRCCS Humanitas Research Hospital, Via Alessandro Manzoni 56, Rozzano, 20089 Milan, Italy; paolo.angeletti@humanitas.it; 3 Scuola di Specializzazione Patologia Clinica e Biochimica Clinica, Bicocca University, 20126 Milan, Italy; paoloangelo.varesini@asst-santipaolocarlo.it; 4 UO of General Surgery, Giuseppe Murri Hospital AST Fermo, Via Augusto Murri, 63900 Fermo, Italy; zuleyka.bianchi@sanita.marche.it; 5 Department of Applied Clinical Sciences and Biotechnology, University of L’Aquila, Via Vetoio, Coppito, 67100 L’Aquila, Italy; francesco.masedu@univaq.it

**Keywords:** colorectal cancer, early detection, colonoscopy, colon capsule endoscopy, computed tomographic colonography, polyps, diagnostic accuracy, systematic review, meta-analysis, network meta-analysis

## Abstract

**Background:** Early detection and prevention of colorectal cancer (CRC) are key goals of population-based screening. Several diagnostic tests have been proposed for CRC screening. This study compares the diagnostic accuracy of colon capsule endoscopy (CCE), colonoscopy (COL) and computed tomographic colonography (CTC), focusing on risk factors such as polyps. **Methods:** We conducted a systematic review with meta-analyses and network meta-analysis. Pooled estimates of sensitivity (SE), specificity (SP), positive predictive value (PPV) and negative predictive value (NPV) were calculated using a random-effects model. Diagnostic performance was assessed for first- and second-level screening based on effect size estimates. **Results:** For first-level screening, sensitivity was 0.79 (95% CI: 0.60–0.91) and specificity 0.95 (95% CI: 0.88–0.98); PPV and NPV were 0.89 and 0.97, respectively. In second-level screening, sensitivity was 0.75 (95% CI: 0.65–0.83), specificity 0.95 (95% CI: 0.92–0.97), PPV 0.76 and NPV 0.95. The indirect sensitivity estimate of CCE vs. COL (SMD = 0.30; 95% CI: 0.12–0.47) was lower than the direct estimate for CTC (SMD = 0.44; 95% CI: 0.29–0.59). CCE showed better comparative performance than CTC relative to COL (CCE SMD = −0.18; 95% CI: −0.29 to −0.06 vs. CTC SMD = −0.98; 95% CI: −1.07 to −0.90). However, both CCE and CTC had lower specificity than COL. **Conclusions:** CCE represents a valuable tool for early CRC detection. Test selection should be guided by clinical and epidemiological settings to optimize screening strategies.

## 1. Introduction

Improvements in the health status of populations and the progressive increase in life expectancy call for the promotion and diffusion of healthy lifestyles, to reduce the impact of non-communicable diseases. Cancer prevention has become one of the most significant public health challenges of the 21st century. In Europe, cancer screening programs are a fundamental pillar of secondary prevention strategies, with well-established recommendations supported by the European Commission [1] for three cancer sites: breast, cervical and colorectal. Colorectal cancer is the third most common cancer worldwide. In 2022, more than 1.9 million cases were diagnosed. Colorectal cancer is the second most common cause of cancer death [2], although effective screening exists that could reduce the number of deaths. Cancer screening is based on two main principles: early detection and prevention [3]. Early detection with screening [4,5] can reduce mortality, and prevention can detect and remove precursor lesions of cancer. Many diagnostic tests have been proposed for cancer screening, such as immunohistochemistry (FIT), high-sensitivity guaiac-based, stool DNA, colon capsule endoscopy (CCE), colon computed tomographic colonography (CTC), flexible sigmoidoscopy and colonoscopy (COL). Each of these has advantages and disadvantages: colonoscopy is the most accurate method, FIT is the easiest to perform, and CT colonography is minimally invasive. The gold standard for CRC screening and adenoma is colonoscopy. However, it should be emphasized that colonoscopy is affected by the operator’s experience [6]. However, there are contraindications to colonoscopy or sedation, which require alternative methods such as the use of CCE [7]. Regarding CCE, it is a modality for CRC screening introduced in 2000 for small-bowel investigation [8,9,10]. The study by Eliakim et al. [10] showed that second-generation CCE had a sensitivity of 89% for the detection of patients with polyps ≥ 6 mm, and for those with polyps ≥ 10 mm, it was 88%, with specificities of 76% and 89%, respectively.

Subsequently, Vuik et al. [11] in their systematic review concluded that CCE appeared to be a safe and effective tool for the detection of CRC and polyps in a screening setting. They also stated that its accuracy was comparable to colonoscopy (COL) and superior to CTC.

Our study aimed to compare the diagnostic tests CCE, COL and CTC with regard to the presence of important colorectal cancer risk factors, such as polyps. Their diagnostic performance was estimated in terms of effect sizes.

## 2. Methods

We conducted systematic literature research in electronic databases, including PubMed, Scopus, Embase and Web of Science. The keywords used for each database are reported in Supplementary Table S1. The literature research was updated on 14 April 2025. Articles published in English in the last 15 years were included. We excluded non-peer-reviewed research such as abstracts, case reports, letters to the editor and reviews. The study selection was conducted by PRISMA methods and finally revised according to the PRISMA Checklist (Supplementary Table S2) [12].

The studies were divided as follows: (i) first-level studies, involving populations undergoing colorectal cancer screening who had not previously undergone any first-line test (fecal occult blood test or colonoscopy), in which PillCam COLON 2 capsule endoscopy was administered as a first-line test; (ii) second-level studies, in which the PillCam COLON 2 capsule was administered after a positive fecal occult blood test, or in a class of patients already referred for endoscopic evaluation due to various clinical reasons (e.g., family history of colorectal cancer, anemia). Two independent reviewers (PMA and PAV) screened titles and abstracts, followed by full-text review for eligible studies. Data were extracted using a pre-specified form, including the following study characteristics: author, year of publication, study design, population characteristics and sample size. SP, SP, PPV, NPV, LR+ and LR were determined. Discrepancies between reviewers were resolved through discussion with a third reviewer (Reviewer EA).

The quality of the included studies was assessed using the QUADAS-2 scale (Supplementary Tables S3 and S4 and Supplementary Figures S1 and S2) [13].

It was impossible to register the systematic review on PROSPERO because the flag “diagnostic test accuracy” was not active.

Given that this study was a meta-analysis of published studies, ethical approval was not required.

### 2.1. Statistical Analysis

#### 2.1.1. Meta-Analysis

Diagnostic accuracy was assessed by calculating sensitivity, specificity, PPV and NPV. The pooled estimates of SE, SP, PPV and NPV were calculated using a random-effects model, if the diagnostic accuracy of the test varied across studies. Heterogeneity among studies was assessed using the I^2^ statistic. Subgroup analyses were performed based on lesion diameter and their characteristics (polyps or adenomas). Forest plots and summary statistics were generated to visualize the pooled diagnostic accuracy. These data were analyzed using Stata 17.

#### 2.1.2. NMA

Within the primary studies considered in this meta-analysis, no one reported a direct comparison between CCE and COL. The lack of head-to-head comparisons makes it important to be able to calculate the difference in diagnostic impact by exploiting an indirect comparison. For this purpose, we used the frequentist network meta-analysis framework (NMA) to provide an estimate of the indirect effect sizes of the CCE and COL diagnostic tests according to the parameters SE, SP, PPV and NPV. The effect sizes were calculated using SMD (standard mean deviation) according to the parameters SE, SP, PPV and NPV.

The estimation of the effect size required the use of a random-effects network meta-analysis model, due to the high heterogeneity between the studies considered.

The $I^{2}=max\left(\frac{Q-df}{Q},0\right)$ statistics were always bigger than 66.4%, as observed when performing the NMA on the sensitivity of the tests. Likewise, the statistical tests of significance on the Q statistics reached small *p*-values (*p* < 0.01). It is known that the assumption of transitivity, i.e., that indirect comparison validly estimates the unobserved head-to-head comparison, cannot be tested statistically, but in our setting, it was plausible, according to Salati et al. [14].

Consistency in our network of tests could not be assessed because we did not have the direct comparison of CCE vs. COL. This is the main reason for using the NMA approach. Formally, we could not test ${\hat{\vartheta }}_{CCE\;Vs\;COL}^{\mathrm{direct}}={\hat{\vartheta }}_{CCE\;Vs\;COL}^{\mathrm{indirect}}$. So, in this study, we shall assume in the first instance that all the direct and indirect effects are consistent with each other and that differences are due to random error.

The use of a frequentist NMA made it possible to suggest the ranking of the tests using P-scores. P-score in our setting ranks tests based on the mean probability of a test being superior to other tests within the network. Higher P-scores represent a higher likelihood of a test being more effective than the others.

The NMA statistical analyses were conducted using the “netmeta and “metar” libraries of the statistical software R (Version 4.5.0).

## 3. Results

**Figure 1:**
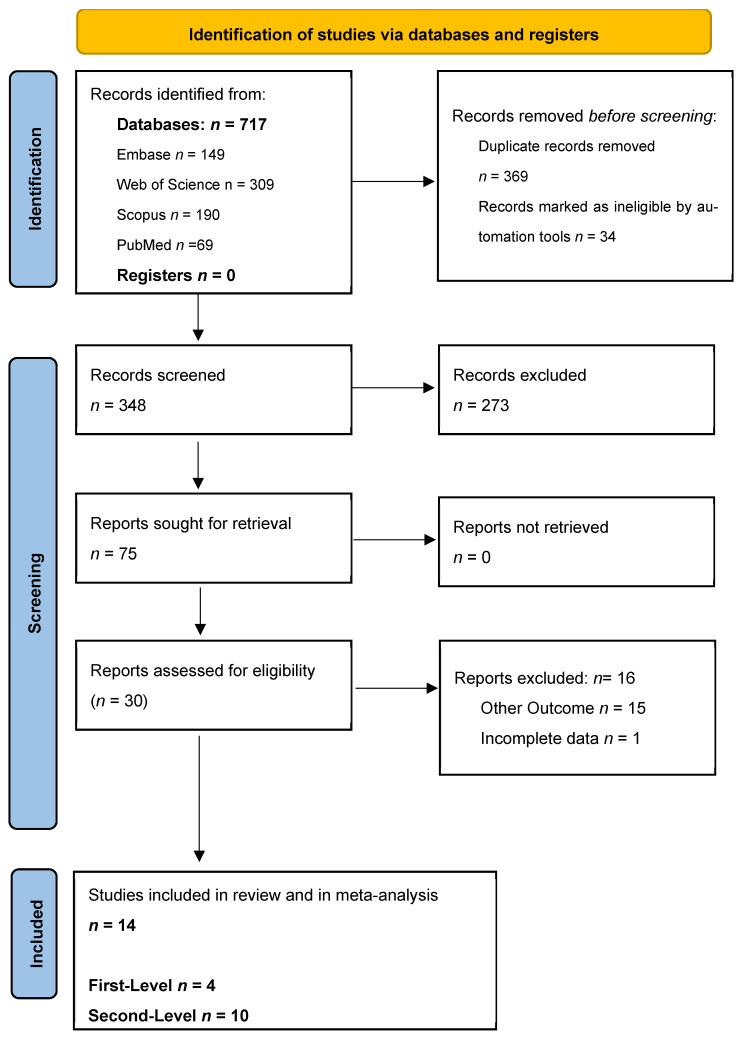
PRSIMA flow-chart results.

A total of 717 records were initially identified through database and register searches. After removing duplicates, 348 records remained for screening. Of these, 273 were excluded based on title and abstract review. The remaining 75 full-text articles were assessed for eligibility, leading to the exclusion of 15 articles for the following reasons: other outcomes (*n* = 16) and incomplete data report (*n* = 1) (Figure 1). Ultimately, 14 studies met the inclusion criteria and were included in the qualitative and quantitative synthesis: 4 at the first diagnostic level (screening in asymptomatic patients) (Table 1) and 10 regarding the second diagnostic level (screening in symptomatic patients or with positive triage test, i.e., fecal occult blood test) (Table 2). The types of bowel preparations employed are provided in Table 3.

Findings concerning publication bias are reported in Supplementary Tables S3 and S4.

## 4. Systematic Review Results

### 4.1. First Level: CCE as a Triage Test in a General Population Undergoing Screening for Colon Cancer

The systematic review of the literature identified four studies in which capsule colonoscopy was used as a first-level test in a general population undergoing screening for colon cancer. All patients underwent capsule examination, followed by colonoscopy (Table 1).

In the study conducted by Rex et al. [15], the authors evaluated the effectiveness of CCE-2 in detecting colorectal polyps among asymptomatic individuals undergoing routine screening. Their prospective study involved 884 asymptomatic subjects aged 50 to 75 years. Participants first underwent CCE-2, followed by conventional colonoscopy (CC). For polyps ≥ 6 mm, SE and SP were 88% and 82%, respectively. For polyps ≥ 10 mm, SE and SP were 92% and 95%, respectively. The study concluded that CCE-2 is a safe and effective non-invasive method for detecting colorectal polyps in an average-risk screening population. It has high sensitivity and specificity, particularly for polyps ≥ 10 mm.

In the study published by Voska et al. [16], the authors evaluated the effectiveness of CCE2 compared to conventional colonoscopy (CC) in detecting colorectal neoplasia. It included 225 asymptomatic individuals aged 50 to 85 who were referred for CRC screening. Participants first underwent CCE2, followed by CC. In terms of polyp detection, CC identified polyps in 114 subjects (51%). Specifically, polyps ≥ 6 mm were found in 34 patients (15%), and polyps ≥ 10 mm in 16 patients (7%). Sensitivity of CCE2 for polyps ≥ 6 mm was 79% (95% CI 62–91). For polyps ≥ 10 mm, SE was 88% (95% CI: 62–98), and for adenomas ≥ 10 mm, SE was 100% (95% CI 72–100).

In the study reported by Pilz et al. [17], the authors evaluated the performance of CCE-2 as a screening tool for CC, comparing its effectiveness to that of conventional colonoscopy, and a total of 59 asymptomatic individuals (34 males and 22 females, median age 59) undergoing routine CC screening were included. Participants underwent CCE on the first day, followed by CC. The overall sensitivity of CCE for detecting significant polyps was 79% (95% CI 61–90), and the specificity was 54% (95% CI 35–70). The PPV of CCE was 63%, and the NPV was 71%.

The TOPAZ study, conducted by Cash et al. [18], compared the diagnostic yield of CCE-2 versus CT colonography (CTC) in a CC screening population. The study enrolled patients aged 50 to 75 years (45 to 75 for African patients) who were at average risk for CC. Participants were randomized to undergo either CCE or CTC, followed by a blinded optical colonoscopy (OC) as the reference standard. For polyps ≥ 6 mm, CCE sensitivity was 79.2% and CCE specificity 96.3%; CTC sensitivity was 26.8% and CTC specificity 98.9%. The TOPAZ study concluded that CCE has a higher diagnostic yield and sensitivity for detecting colorectal polyps ≥6 mm compared to CTC. These findings suggest that CCE may be a more effective non-invasive screening option for colorectal cancer in average-risk populations.

### 4.2. Second-Level Evaluation: Diagnostic Accuracy of CCE in FIT-Positive or High-Risk Populations

A review of selected studies highlights the diagnostic performance of colon capsule endoscopy (CCE) in second-level screening scenarios, including patients with a positive fecal immunochemical test (FIT) or clinical indications suggestive of colonic disease.

Gonzales-Suarez et al. [19] published an RCT of 286 patients (mean age 59), where CCE showed excellent diagnostic accuracy. For lesions ≥ 6 mm, sensitivity was 96%, specificity 88.2%, PPV 90.2% and NPV 95.2%. For lesions ≥ 10 mm, sensitivity reached 97.3% and specificity 95.3%, with an AUC of 95.5. These results were comparable or superior to CT colonography, especially for large neoplastic lesions.

In a smaller RTC of 78 patients by Pioche et al. [20], diagnostic accuracy was markedly lower, with sensitivity and specificity of 60.0% and 40.0%, respectively, for lesions > 9 mm. CT colonography also showed limited performance (sensitivity 28.6%, specificity 53.6%). Rondonotti et al. [21] conducted a prospective cohort study of 50 patients (mean age 59.2). CCE achieved good sensitivity and specificity for polyps 6–9 mm (88.2% and 87.8%) and lesions > 9 mm (92.8% and 91.6%). Diagnostic likelihood ratios confirmed high rule-in and rule-out capacity (LR+ > 4, LR– < 0.1).

In the study conducted by Holleran et al. [22], among 62 patients (mean age 62.5), CCE showed very high sensitivity for lesions > 9 mm (97%) and a specificity of 86%, with high PPV and NPV (97% and 86%, respectively) and an LR+ of 23.11, indicating strong discriminative performance. Pecere et al. [23] demonstrated moderate-to-high diagnostic values in 222 subjects: For 6–9 mm adenomas, sensitivity was 90.0%, but specificity was lower (66.1%). For lesions >9 mm, sensitivity and specificity were 76.7% and 90.7%, respectively, with corresponding LR+ and LR– values indicative of reasonable rule-in ability.

Kobaek-Larsen et al. [24] demonstrated in 253 patients that CCE has robust diagnostic values for polyps > 9 mm, with sensitivity of 87%, specificity of 92% and LR+ of 10.71. These findings support its use in advanced lesion detection.

### 4.3. Mixed-Population Studies

In Spada et al. [25], in 117 patients, CCE showed lower diagnostic performance for 6–9 mm lesions (SE 84%, SP 64%), but this significantly improved for lesions > 9 mm (SE 88%, SP 95%). Notably, NPV remained high across size categories. In Eliakim et al. [9], among 98 patients (mean age 49.8), CCE showed consistent sensitivity (SE 89% for 6–9 mm, 88% for >9 mm) and moderate specificity. NPV was high (97%), though PPV was lower, especially in small lesions. In Morgan et al. [26], CCE yielded high sensitivity for 6–9 mm lesions (93.3%) and perfect sensitivity for lesions > 9 mm (100%), with excellent NPV (97–98%), supporting its potential in small-cohort settings. Finally, in Parodi et al. [27], CCE showed strong diagnostic accuracy: SE and SP for 6–9 mm were 91% and 88%, and for lesions > 9 mm, they were 89% and 95%, respectively. Likelihood ratios further confirmed the method’s diagnostic value (LR+ up to 16.7; LR– as low as 0.12).

## 5. Colon Capsule as First-Level Diagnostic Test: Meta-Analysis Results

In the context of first-level screening, the overall analysis showed a sensitivity of 0.79 (95% CI: 0.60–0.91; I^2^ = 57.59) and a specificity of 0.95 (95% CI: 0.88–0.98; I^2^ = 45.86). The positive predictive value (PPV) was 0.89, while the negative predictive value (NPV) was 0.97, with considerable heterogeneity across studies (I^2^ = 94.9%).

### 5.1. Adenomas 6–9 mm

For intermediate-sized adenomas (6–9 mm), the sensitivity was 0.83 (95% CI: 0.76–0.88; I^2^ = 26.82), and the specificity was 0.95 (95% CI: 0.93–0.97; I^2^ = 30.21). The PPV was also 0.95, while the NPV was 0.86 (I^2^ = 81.98), suggesting high diagnostic performance with moderate-to-high heterogeneity in NPV estimates.

### 5.2. Adenomas >9 mm

In lesions >9 mm, diagnostic accuracy was optimal, with a sensitivity of 0.85 (95% CI: 0.78–0.90; I^2^ = 0.00) and a specificity of 0.98 (95% CI: 0.97–0.99; I^2^ = 0.00). The PPV was 0.91, and the NPV was 0.95 (I^2^ = 71.55), indicating excellent performance with consistent results across studies.

### 5.3. Polyps 6–9 mm

For polyps of similar dimensions, sensitivity was very high (0.95, 95% CI: 0.86–0.98), and specificity reached 0.97 (95% CI: 0.94–0.99). The PPV was 0.86. NPV was not fully reported for this subgroup.

## 6. Second-Level Testing—Overall Analysis

Second-level testing showed slightly lower diagnostic performance. The overall sensitivity was 0.75 (95% CI: 0.65–0.83; I^2^ = 75.00), with a specificity of 0.95 (95% CI: 0.92–0.97; I^2^ = 55.41). PPV and NPV were 0.76 and 0.95, respectively, with marked heterogeneity for NPV (I^2^ = 98.4%).

### 6.1. Polyps and Adenomas 6–9 mm

In this subgroup, sensitivity was 0.69 (95% CI: 0.55–0.80; I^2^ = 75.87), and specificity remained high at 0.95 (95% CI: 0.92–0.97; I^2^ = 20.93). The PPV was relatively low (0.63), while the NPV was 0.95, again with high heterogeneity (I^2^ = 96.8).

### 6.2. Polyps and Adenomas >9 mm

Lesions larger than 9 mm showed a sensitivity of 0.79 (95% CI: 0.67–0.88; I^2^ = 72.40) and a specificity of 0.94 (95% CI: 0.89–0.97; I^2^ = 63.56). The PPV and NPV were 0.79 and 0.96, respectively (I^2^ = 98.8), confirming good diagnostic accuracy for advanced lesions.

### 6.3. FIT-Positive Population

Among patients with a positive FIT result, sensitivity was 0.81 (95% CI: 0.69–0.89; I^2^ = 76.54), and specificity was 0.92 (95% CI: 0.86–0.96; I^2^ = 61.87). PPV and NPV were 0.81 and 0.94, respectively, with high heterogeneity for NPV (I^2^ = 99.7).

### 6.4. Mixed Cases

**Figure 2:**
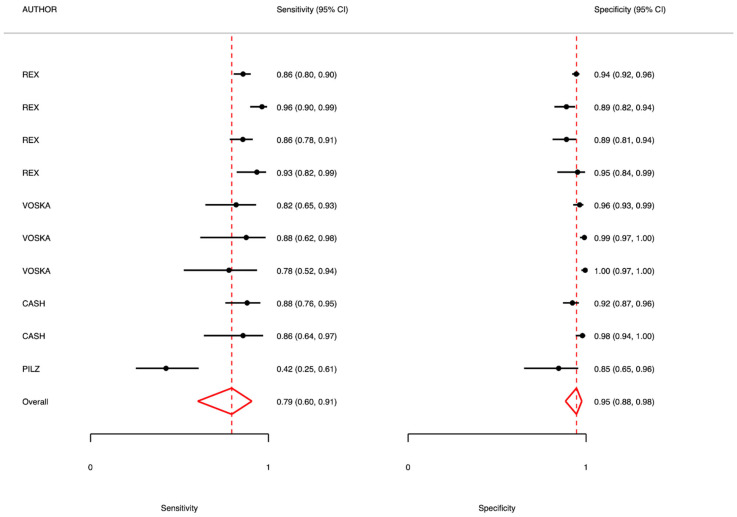
Meta-analysis results: CCE usage in first-level screening of general population compared to gold standard (colonoscopy).

**Figure 3:**
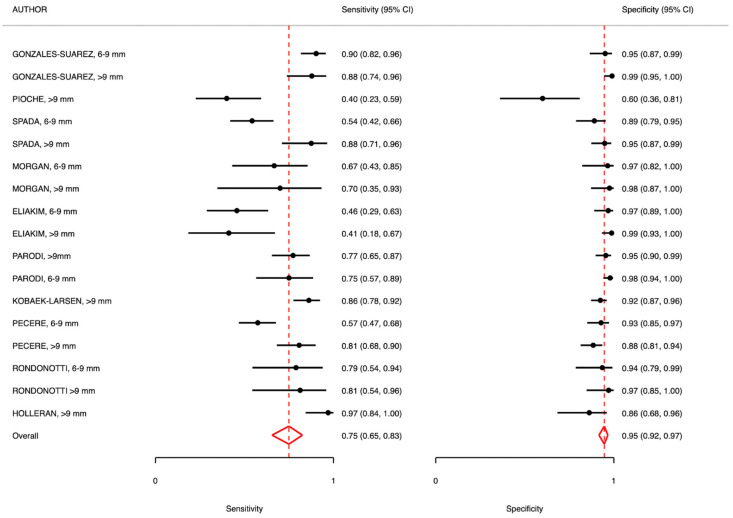
Meta-analysis results: CCE usage in second-level screening of FOBT+ patients and/or average-risk patients (familiarity, genetic mutation, anemization) compared to gold standard (colonoscopy).

In studies including mixed populations, sensitivity was lower (0.66, 95% CI: 0.54–0.76; I^2^ = 63.68), whereas specificity remained high at 0.96 (95% CI: 0.94–0.98; I^2^ = 23.84). The PPV was 0.70, and the NPV was 0.96, also showing substantial heterogeneity (I^2^ = 97.3) (Table 4 and Figure 2 and Figure 3).

The NMA effect size estimations (Table 5) using random-effects models show statistically significant SMD for SE and SP as shown by the 95% CI for CCE vs. COL and the indirect comparison, as well as for CTC. PPV and NPV do not reach statistically significant results, having wide CIs for both the test parameters. The indirect CCE sensitivity estimate of SMD compared to COL (0.30 (0.12; 0.47)) yields a lower value than the direct estimate of the CTC sensitivity (0.44 (0.29; 0.59)). The performance changes according to the specificity of CCE and CTC tests. They both reach a statistically significant estimate, but the analysis suggests that CCE (−0.18 (−0.29; −0.06)) performs better with respect to COL than CTC (−0.98 (−1.07; −0.90)). In terms of specificity, both CCE and CTC underperform with respect to COL. These estimates suggest different uses of the tests according to the clinical and epidemiological settings.

Based on the results of the network meta-analysis, P-scores (Table 6) were calculated to rank the diagnostic performance of the three evaluated modalities—COL, CCE and CTC—across four key parameters: sensitivity (Se), specificity (Sp), positive predictive value (PPV) and negative predictive value (NPV). COL achieved the highest P-scores for specificity (0.9999), PPV (0.9969) and NPV (*p* = 0.8655), indicating a strong performance, as would be expected from a gold standard test. Among other diagnostic tests, CCE demonstrated the highest P-score with respect to CTC.

## 7. Discussion

An ideal screening test should combine high diagnostic accuracy with solid practical applicability in public health settings. From the accuracy point of view, the test should have a high sensitivity, to minimize false negatives and ensure early identification of subjects affected by the disease, and a good specificity, to avoid false positives [28]. Fecal immunochemical testing (FIT) is currently the most recommended method in organized colorectal cancer screening programs in Europe. This is due to its favorable balance between diagnostic performance, acceptability and cost-effectiveness, with different organizational layouts across European countries [5]. Average sensitivity values of FIT for colorectal cancer range between 73% and 88%, while for advanced precancerous lesions (advanced adenomas), sensitivity varies between 22% and 40%, depending on the cut-off used for fecal hemoglobin [29]. FIT specificity is generally high, with values exceeding 90%, ensuring a low false positive rate and therefore good efficiency in selective referral for colonoscopy [30]. However, the effectiveness of a test is not limited to its intrinsic properties. It must also be easily accessible, acceptable to the target population, reproducible, and sustainable in economic and organizational terms [31]. A test that is too invasive or expensive, even if accurate, may fail to achieve adequate coverage, nullifying the potential benefit of screening. The balance between diagnostic performance and operational feasibility is therefore the cornerstone for the effective adoption of a screening test in a public health program.

Colonoscopy, considered the gold standard for the diagnosis of colorectal cancer, has sensitivity and specificity values close to 95–100% for advanced tumors and adenomas [32]. In addition, colonoscopy allows the simultaneous identification and removal of precancerous lesions, providing not only a diagnosis but also immediate therapeutic intervention. However, its accuracy may vary based on technical and operator factors, such as the quality of the bowel preparation and the experience of the endoscopist. Despite its excellent diagnostic performance, some other limitations of colonoscopy include the following: its applicability, invasiveness, need for sedation, procedural risks (such as perforation or bleeding, although rare), and lower acceptability by the population compared to FIT.

To the best of our knowledge, this is the first network meta-analysis directly comparing colon capsule endoscopy (CCE), conventional colonoscopy (COL) and CT colonography (CTC) for the detection of colonic polyps. Our findings align with previous evidence, such as the systematic review by Vuik et al. [11], but expand upon them by incorporating a broader and more recent set of studies, including those published during and after the COVID-19 pandemic. The overall results of this meta-analysis indicate that capsule colonoscopy may represent, like self-sampling or mammography performed “on the road”, a possibility of access to screening for patients with a positive FIT, who refuse a colonoscopy for further diagnostic investigation. Also, it proves useful in those patients in whom there is a high probability of false positives due, for example, to the presence of hemorrhoidal pathology or congenital or iatrogenic coagulation alterations or in patients with easy bleeding such as those taking anticoagulant and aggregating drugs. The first colonoscopy capsule (PillCam COLON) was approved for clinical use in Europe in the late 2000s. The second generation, PillCam COLON 2, significantly improved diagnostic performance, thanks to a wider field of view, higher image resolution and an acquisition speed control system based on intestinal movement [25]. Our meta-analysis confirms that colon capsule endoscopy (CCE) can represent a valid complementary option, especially for patients who refuse colonoscopy.

A recent study conducted n the UK by Turvill et al [33], in which the capsule was used on a large scale during the pandemic, highlighted how the capsule is an excellent diagnostic tool. CCE identified all carcinomas in patients with a complete examination and well-prepared intestine. Sensitivity was 97% for polyps > 9 mm and 6–9 mm, detecting overall more polyps ≥ 6 mm compared to colonoscopy and CTC. CCE was well tolerated in 98.4% of patients, and only 0.2% had problems related to capsule retention. A strong limitation was the overall preparation of the colon, since only in 63% of cases was it adequate, altering the overall diagnostic performance. The study also evaluated how CCE reduced urgent colonoscopies by 86% [33].

A cost analysis conducted in 2024 by the Scottish Health Service demonstrated that CCE is cost-effective for diagnostic and surveillance purposes in symptomatic patients who decline colonoscopy. However, the economic efficiency of the procedure decreases markedly when applied to the general screening population [34]. As noted by Baatrup et al. [35], CCE is unlikely to remain cost-effective if the reinvestigation rate—defined as the proportion of patients requiring follow-up colonoscopy—exceeds 30%. In clinical practice, reinvestigation rates as high as 70% have been reported, leading to a doubling of overall diagnostic costs and substantially diminishing the viability of CCE as a first-line strategy [33]. These findings underscore the importance of restricting CCE utilization to clearly defined subgroups of patients, particularly those who refuse colonoscopy or present with clinical contraindications to invasive procedures.

## 8. Limitations

Our meta-analysis has some limitations. First, there was high heterogeneity observed in the statistical analysis. This may be due to the different bowel preparation protocols used across studies, the heterogeneity of the study populations included and the variability in image reading protocols and software, across 15 years of published studies.

Such differences could have impacted diagnostic accuracy and explain some of the variation observed across studies in the final analysis. Finally, while this is the first network meta-analysis comparing CCE, colonoscopy and CT colonography for the detection of colorectal polyps, the number of direct head-to-head trials remains limited. Many comparisons rely on indirect evidence, which introduces some uncertainty. Future large-scale, prospective studies using standardized bowel preparation and image analysis protocols, and including economic endpoints, are warranted to validate these findings.

## 9. Conclusions

The data derived from our meta-analysis shows that there are no substantial differences between first- and second-level screening, thus supporting the hypothesis of administering the capsule in certain circumstances, such as diagnostic investigation in patients with FIT+ or with known familiarity.

The statistical heterogeneity found may be the result of different forms of administration, linked to problems inherent in intestinal cleansing or analysis and interpretation of the acquired images.

Finally, although the choices surrounding bowel preparation are not within the scope of this work, and although other large case studies show how this aspect can affect the overall performance of population screening [36], our data confirm that the capsule can be an excellent early diagnosis tool, to be used in various healthcare settings, to increase early diagnosis of colorectal cancer.

## Figures and Tables

**Table 1:** Studies on first-level tests in a general population undergoing screening for colorectal cancer.

Author, Year, Country	Study Population			Results		
	Study Setting	Sample Size, Gender (% Males), Age	Outcome	Diagnostic Accuracy: Se, Sp, VPN, VPP, LR+LR−		Other Results
				Colon Capsule		
				Dimension		
				6–9 mm	>9 mm	
Rex 2015 USA [15]	Prospective cohort study	885 44% male mean age 57 y. range not reported	Polyps and Adenomas	Polyps SE 88 (82–93) SP 82 (80–83) PPV 86 (83–88) NPV 95 (94–96) LR+ 14.5 LR− 0.14 Adenomas SE 81 (77–84) SP 93 (91–95) PPV 85 (82–89) NPV 89 (86–93) LR+ 5.35 LR− 0.11	Polyps SE 92 (82–97) SP 95 (94–95) PPV 96 (93–98) NPV 89 (86–92) LR+ 28.33 LR− 0.15 Adenomas SE 80 (74–86) SP 97 (96–98) PPV 93 (89–97) NPV 95 (91–98) LR+ 11.88 LR− 0.05	Colonoscopy result frequency (%) Polyps 6–9 mm 28% >9 mm 11% Adenomas 6–9 mm 16% >9 mm 6%
Voska 2019 Czech Republic [16]	Prospective cohort study	225 men, 53% mean age: 59 years; range: 50–81 years	Polyps and Adenomas	Polyps SE 79 (62–91) SP 97 (80–83) PPV 82 (75–90) NPV 96 (95–98) LR+ 26.34 LR− 0.22	Polyps SE 88 (62–98) SP 97 (80–83) PPV 87 (75–90) NPV 99 (99–100) LR+ 88 LR− 0.12 Adenomas SE 100 SP 98 (80–83) PPV 76 (65–88) NPV 100 (100–100) LR+ 50 LR− 0	Colonoscopy result frequency (%) Polyps 6–9 mm 15% >9.7% Adenomas >9.5%
Pilz 2010 Switzerland [17]	Prospective cohort study	59	Polyps and Adenomas	SE 79 (61–90) SP 54 (35–70) PPV 71 (62–80) NPV 66 (54–79) LR+ 2.08 LR− 0.65		Colonoscopy result frequency (%) 50% (*n* = 28)
Cash 2020 USA [18]	Prospective randomized study	320	Polyps	Polyps 6–9 mm SE 79.2 (66.4–82.2) SP 96.3 (89.1–92.2) PPV 88 (83–93) NPV 93 (90–95) LR+ 19.75 LR− 0.22 Polyps >9 mm SE 85.7 (64.5–95.9) SP 98.2 (93.3–99.0) PPV 86 (78–94) NPV 98 (97–99) LR+ 43 LR− 0.14	Adenomas not reported	Colonoscopy result frequency (%) Polyps 6–9 mm 27.6% Polyps > 9 mm 10.3% Adenomas 6–9 mm 17.6% Adenomas > 10 mm 6.1%

**Table 2:** Studies according to diagnostic level: screening in symptomatic patients or positive fecal occult blood test.

Author, Year, Country	Study Population			Main Results		Other Results
	Study Setting	Sample Size	Outcome	Diagnostic Accuracy		
	FIT+					
Gonzales-Suarez Spain 2020 [19]	RCT	*n* = 286 mean age 59 y (50–81 y) men 53%	Polyps and adenomas	6 mm (any neoplastic lesion) SE 96. (91.1–100) SP 88.2 (79.6–95.3) PPV 90.2 (83.5–96.1) NPV 95.2 (89.2–100) AUC 92.4 (87.5–96.5) LR+ 8.04 LR− 0.04	>9 mm (any neoplastic lesion) SE 97.3 (91.1–100) SP 95.3 (90.7–99.0) PPV 87.8 (76.7–97.3) NPV 99.0 (96.8–100) AUC 95.5 (92.4–99.3) LR+ 20.4 LR− 0.02	CT colonoscopy 6 mm (any neoplastic lesion) SE 79.3 (68.6–88.8) SP 96.3 (91.1–100) PPV 93.8 (85.7–100) NPV 86.9 (80.0–93.2) AUC 89.3 (83.6–93.6) 10 mm (any neoplastic lesion) SE 90.0 (83.9–93.9) SP 99.0 (95.6–100) PPV 97.3 (93.1–100) NPV 96.1 (91.5–98.2) AUC 96.4 (91.9–98.4)
Pioche 2018 France [20]	RCT	*n* = 78 mean age and gender not reported on effective performed exams	Adenomas and cancers	>9 mm (any neoplastic lesion) SE: 60.0 (36.1–80.9) SP 40.0 (19.1–63.9) PPV 80.0 NPV 20.0 LR+ 1.0 LR− 1.0		CT colonography SE 28.6 (11.3–52.2) SP 53.6 (33.9–72.5) PPV: 46.4 NPV: 53.6
Rondonotti 2014 Italy [21]	Prospective cohort study	*n* = 50 mean age 59.2 +5.8 y male 58%	Polyps	6–9 mm SE 88.2 (62.2–97.9) SP 87.8 (70.8–96.0) PPV 79 (73–81) NPV 94 (90–94) LR+ 3.7 (1.52–9.2) LR− 0.06 (0.01–0.26)	≥9 mm SE 92.8 (64.1–99.6) SP 91.6 (76.4–97.8) PPV 81 (76–83) NPV 0.97 (0.95–0.98) LR+ 4.3 (1.5–12.3) LR− 0.03 (0.004–0.20)	CT colonography 6–9 mm SE 88.2 (62.2–97.9) SP84.8 (67.3–94.3) LR+ 3.0 (1.34–6.67) LR− 0.07 (0.01–0.27) ≥9 mm SE 78.6 (48.8–94.3) SP 91.7 (76.4–97.8) LR+ 3.7 (1.3–10.4) LR− 0.09 (0.03–0.20)
Holleran Ireland 2013 [22]	Prospective cohort study	*n* = 62 mean age 62.5 + 5.8 y. male 55%	Polyps	≥9 mm SE 97 (84–100) SP 86 (0.68–0.96) PPV 97 (95–99) NPV 86 (82–87) LR+ 23.11 LR− 0.11		Colonoscopy results on significant lesions were the same as CCE
Pecere Italy 2019 [23]	Prospective cohort study	*n* = 222 mean age 66 y. male 56.3%	Adenomas	6–9 mm SE 90.0 SP 66.1 PPV 57 (54–58) NPV 92.9 (91–93) LR+ 2.6 LR− 0.15	≥9 mm SE 76.7 SP 90.7 PPV 80.7 (78–82) NPV 88.4 (86–89) LR+ 8.2 LR− 0.25	
Kobaek-Larsen 2018 [24]	Prospective cohort study	*n* = 253	Polyps	>9 mm SE 87 (78–91) SP 92 (89–95) PPV 86 (84–87) NPV 92 (91–93) LR+ 10.71 LR− 0.14		
**MIXED CASES**						
Spada 2010 Italy [25]	Prospective cohort study	*n* = 117 mean age 60 +9 y male 61.5%	Neoplasia and adenomas	6–9 mm SE 84 (74–95) SP 64 (52–76) PPV 54 (50–55) NPV 89 (86–89) LR+ 2.3 LR− 0.24	>9 mm SE 88 (76–99) SP 95 (90–100) PPV 88 (84–88) NPV 95 (93–95) LR+ 17.06 LR− 0.13	6–9 mm 41% ≥9 mm 29%
Eliakim 2009 Israel [10]	Prospective cohort study	*n* = 104 mean age 49.8 (range 18–57)		6–9 mm Se 89 (70–97) Sp 76 (72–78 PPV 46 (41–46) NPV 97 (95–97) LR+ 3.7 LR− 0.14	>9 mm SE 88 (56–98) SP 89 (86–90) PPV 41 (36–46) NPV 97 (95–97) LR+ 7.8 LR− 0.14	6–9 mm 19% ≥9 mm 8%
Morgan USA 2016 [26]	Prospective cohort study	*n* = 50 mean age 60.2 y (range 32–70) male 45%	Polyps	6–9 MM SE 93.3 (66.0–97.7) SP 80 (62.5–90.9) PPV 67 (60–68) NPV 97 (94–97) LR+ 4.6 LR− 0.08	>9 mm SE 100 (56.1–100.0) SP 93.0 (79.9–98.2) PPV 70 (64–71) NPV 98 (95–98) LR+ 12.5 LR− 0.13	6–9 mm 30% ≥9 mm 14%
Parodi Italy 2017 [27]	Prospective cohort study	*n* = 177 mean age 57 y. (26–82) male 45.3%	Polyps	6–9 mm SE 91 (81–96) SP 88 (81–93) PPV 75 (72–75) NPV 98 (97–98) LR+ 16.7 LR− 0.17	>9 mm SE 89 (72–96) SP 95 (90–97) PPV 77 (74–78) NPV 95 (94–96) LR+ 7.34 LR− 0.12	OC diagnosed 243 lesions, of which 151 (62.1%) < 6 mm, 92 (37.9%) ≥ 6 mm and 41 (16.9%) ≥ 10 mm

**Table 3:** Bowel preparation protocols.

Author	Bowel Preparation Method
Rex [15]	Senna (12 mg × 4 tablets, 2 days prior), clear liquids (day before), 2 L PEG-ELS (evening + morning), oral sulfate solution (SUPREP), bisacodyl if delayed capsule transit.
Voska [16]	PEG with sodium phosphate booster (details limited).
Pilz [17]	Low-fiber diet (3 days), 4 L PEG (Cololyt^®^), domperidone, sodium phosphate (Colophos^®^) boosters, bisacodyl suppository.
Cash [18]	Split-dose PEG-based regimen; details in Supplementary Materials.
Gonzales-Suarez [19]	Low-residue diet for 3 days prior. Day -1: 2 L PEG (PM). Day 0: 2 L PEG (AM), then capsule ingestion. Booster 1: 40 mL sodium phosphate + 1 L water. Booster 2 (if no excretion after 2 h): 20 mL sodium phosphate + 1 L water.
Pioche [20]	2-day prep: Day -2: 4 L PEG + water; Day -1: 3 L PEG + clear liquids; Day 0: 1 L PEG + domperidone + sodium phosphate (55 mL) + bisacodyl suppository.
Rondonotti [21]	Day -3/-2: low-fiber diet; Day -1: clear liquids + Macrogol 3350 + ascorbic acid (2 L), bisacodyl 20 mg at night; Day 0: Macrogol 3350 + ascorbic acid (1 L) before capsule, then metoclopramide IV, NaP booster (30 mL + 1 L), then 15 mL + 500 mL.
Holleran [22]	PEG-based regimen (not fully specified).
Pecere [23]	Split-dose PEG + sodium phosphate and gastrografin boosters.
Kobaek-Larsen [24]	Day -2: 1000 mg magnesium oxide + 2 L water (AM), 1000 mg magnesium oxide (PM). Day -1: clear liquids, 1 L Moviprep + 2 L water (PM). Day 0: 1 L Moviprep + 1 L water (AM), 20 mg domperidone orally. Booster 1: ¾ L Moviprep + 1 L water. Booster 2: ¼ L Moviprep + ¼ L water, 10 mg bisacodyl suppository if no excretion.
Spada [25]	Senna (4 tablets, 2 days prior), 4 L split PEG, sodium phosphate boosters guided by data recorder, bisacodyl suppository if capsule not expelled.
Eliakim [9]	PEG, sodium phosphate, bisacodyl suppositories.
Morgan [26]	Magnesium citrate as booster—shown to result in inadequate cleansing.
Parodi [27]	Day -1: 2 L PEG–electrolyte solution (PM). Day 0: 2 L PEG at 6:00 AM, capsule ingestion after 10 h fasting. Booster 1: 40 mL sodium phosphate + 125 mL water + 1 L water. Booster 2 (after 2 h): 20 mL sodium phosphate + 125 mL water + 1 L water. If no excretion: 10 mg bisacodyl suppository.

**Table 4:** Meta-analysis results.

	Sensibility	Specificity	PPV	NPV
First-level studies				
Overall lesions K= 7	0.79 (0.60–0.91) I^2^ = 57.59	0.95 (0.88–0.98) I^2^ = 45.86	0.89 (0.82–0.91) I^2^ = 93.3%	0.97 (0.96–0.97) I^2^ = 94.9
Adenomas 6–9 mm K = 3	0.83 (0.76–0.88) I^2^ = 26.82	0.95 (0.93–0.97) I^2^ = 30.21	0.95 (0.93–0.97) I^2^ = 81.98	0.86 (0.84–0.88) I^2^ = 0.05
Adenomas >9 K = 3	0.85 (0.78–0.90) I^2^ = 0.00	0.98 (0.97–0.99) I^2^ = 0.00	0.91 (0.84–0.98) I^2^ = 71.55	0.95 (0.89–1.02) I^2^ = 98.53
Polyps 6–9 mm K = 2	0.95 (0.86–0.98)	0.97 (0.94–0.99)	0.86 (0.70–1.01)	-
**Second-level studies**				
Overall lesions K = 17	0.75 (0.65–0.83) I^2^ = 75.00	0.95 (0.92–0.97) I^2^ = 55.41	0.76 (0.73–0.74) I^2^ = 99.5	0.95 (0.95–0.62) I2 = 98.4
Polyps and adenomas 6–9 mm K = 7	0.69 (0.55–0.80) I^2^ = 75.87	0.95 (0.92–0.97) I^2^ = 20.93	0.63 (0.68–0.70) I^2^ = 99.5	0.95 (0.95–0.96) I^2^ = 96.8
Polyps and adenomas >9 mm K = 10	0.79 (0.67–0.88) I^2^ = 72.40	0.94 (0.89–0.97) I^2^ = 63.56	0.79 (0.78–0.80) I^2^ = 99.4	0.96 (0.95–0.97) I^2^ = 98.8
FIT+ K = 6	0.81 (0.69–0.89) I^2^ = 76.54	0.92 (0.86–0.96) I^2^ = 61.87	0.81 (0.80–0.81) I^2^ = 98.5	0.94 (0.94–0.95) I^2^ = 99.7
Mixed cases K = 4	0.66 (0.54–0.76) I^2^ = 63.68	0.96 (0.94–0.98) I^2^ = 23.84	0.70 (0.69–0.71) I^2^ = 99.6	0.96 (0.95–0.97) I^2^ = 97.3

**Table 5:** Effect size estimates using SMD: diagnostic test vs. baseline (COL).

	Baseline (COL)
CCE^Indirect^	SMD_SE_ = 0.30 (0.12; 0.47) SMD_SP_ = −0.18 (−0.29; −0.06) SMD_PPV_ = 0.02 (−0.13; 0.17) SMD_NPV_ = 0.16 (−0.09; 0.40)
CTC^Direct^	SMD_SE_ = 0.44 (0.29; 0.59) SMD_SP_ = −0.98 (−1.07; −0.90) SMD_PPV_ = −0.02 (−0.14; 0.10) SMD_NPV_ = 0.10 (−0.10; 0.31)

**Table 6:** The ranking between tests using the P-score: a higher p-score indicates better diagnostic performance.

	Se	Sp	VPP	VPN
COL	0.9998	1.0000	0.7122	0.8655
CCF	0.1989	0.1901	0.4805	0.1773
CTC	0.0013	0.0006	0.3073	0.1572

## Data Availability

Not applicable.

## Supplementary Materials

The following supporting information can be downloaded at: https://www.mdpi.com/article/10.3390/diagnostics15172157/s1, Table S1: Literature research results. Table S2: Prisma Cheklist. Tables S3 and S4: ROB according to QUADAS-2 scale. Figures S1 and S2: ROB according to QUADAS-2 scale.
